# The Vigilance Decrement in Executive Function Is Attenuated When Individual Chronotypes Perform at Their Optimal Time of Day

**DOI:** 10.1371/journal.pone.0088820

**Published:** 2014-02-19

**Authors:** Tania Lara, Juan Antonio Madrid, Ángel Correa

**Affiliations:** 1 Centro de Investigación Mente, Cerebro y Comportamiento, Universidad de Granada, Granada, Spain; 2 Departamento de Fisiología, Universidad de Murcia, Murcia, Spain; 3 Departamento de Psicología Experimental, Universidad de Granada, Granada, Spain; CEA.DSV.I2BM.NeuroSpin, France

## Abstract

Time of day modulates our cognitive functions, especially those related to executive control, such as the ability to inhibit inappropriate responses. However, the impact of individual differences in time of day preferences (i.e. morning vs. evening chronotype) had not been considered by most studies. It was also unclear whether the vigilance decrement (impaired performance with time on task) depends on both time of day and chronotype. In this study, morning-type and evening-type participants performed a task measuring vigilance and response inhibition (the Sustained Attention to Response Task, SART) in morning and evening sessions. The results showed that the vigilance decrement in inhibitory performance was accentuated at non-optimal as compared to optimal times of day. In the morning-type group, inhibition performance decreased linearly with time on task only in the evening session, whereas in the morning session it remained more accurate and stable over time. In contrast, inhibition performance in the evening-type group showed a linear vigilance decrement in the morning session, whereas in the evening session the vigilance decrement was attenuated, following a quadratic trend. Our findings imply that the negative effects of time on task in executive control can be prevented by scheduling cognitive tasks at the optimal time of day according to specific circadian profiles of individuals. Therefore, time of day and chronotype influences should be considered in research and clinical studies as well as real-word situations demanding executive control for response inhibition.

## Introduction

Maintaining attention to the task at hand over an extended time period (i.e., vigilance) can be crucial in many situations. Research on vigilance has reported a drop-off in performance as time on task increases, the so-called vigilance decrement [1]. The vigilance decrement has been explained in terms of either reduced arousal or depletion of cognitive resources over time [2].

The vigilance level of individuals also fluctuates at longer timescales, for example over the course of the day, as shown by research using the Psychomotor Vigilance Test (PVT) [3]. Time of day further influences higher-order cognitive functions, as indexed by behavioural and neural measures related to executive control [4]–[10].

**Figure 1:**
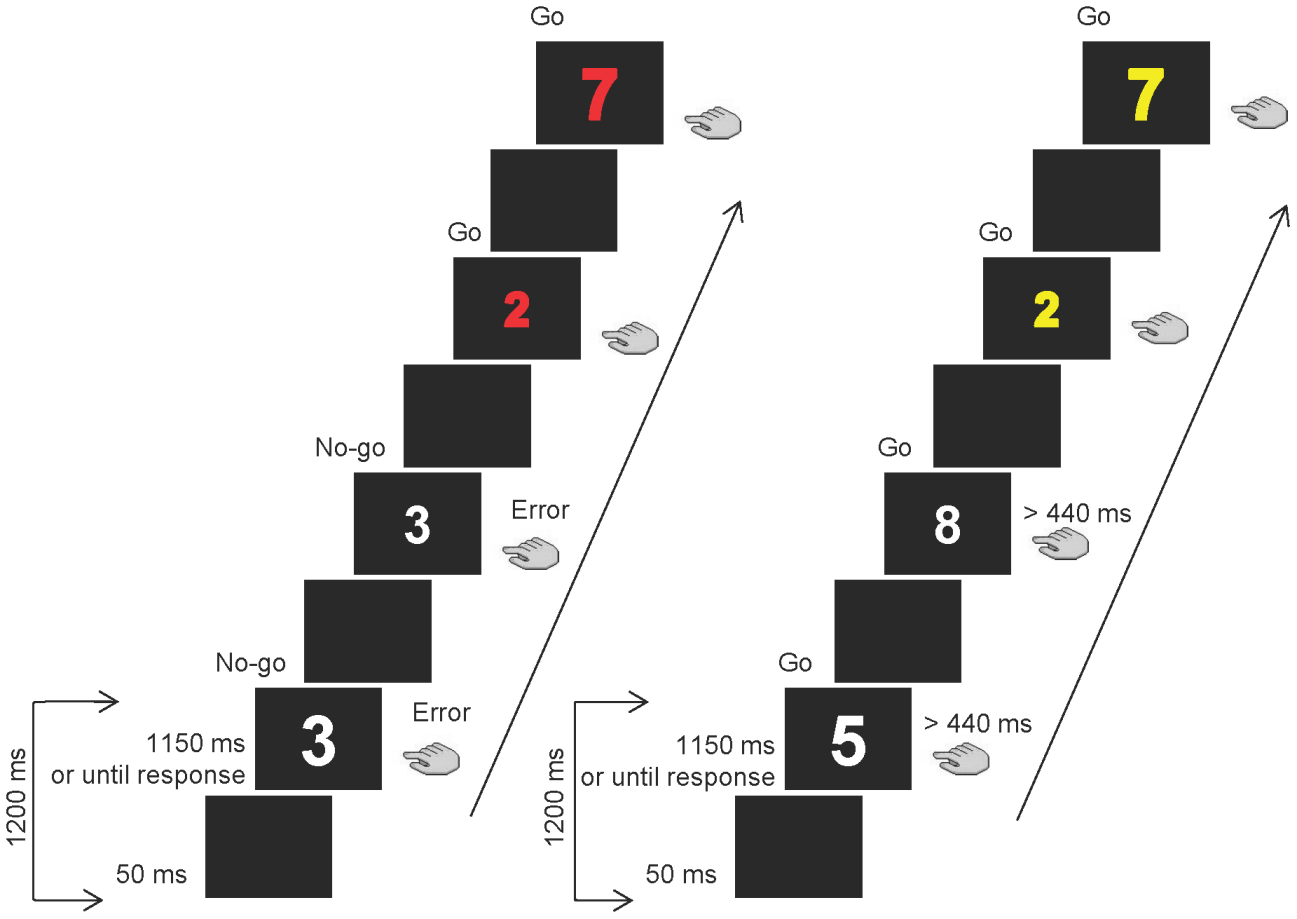
Sequence of events for both strategy conditions in the modified SART. The precision strategy condition, on the left, emphasised accurate response inhibition over fast responding. Digits turned red when the average correct response rate in no-go trials was below 0.71. The speed strategy condition, on the right, emphasised fast over correctly inhibited responses. Digits were presented in yellow when the average RT was above 440 ms and accuracy rate in no-go trials was not below 0.45.

Executive control is typically engaged in novel or complex situations to adapt our behaviour for optimal performance (e.g. inhibiting routine responses when they are inappropriate) [11]. The Sustained Attention to Response Task (SART) [12] measures the ability to sustain executive control for response inhibition over a given period of time. The SART requires fast responses to random single digits from 1 to 9 (go digit), except for the ‘3’ stimulus (no-go digit), to which participants must not respond (see Figure 1 for an example). Therefore, successful response inhibition to infrequent no-go trials demands prolonged attention during task [12].

Grier and colleagues [13] noted that performance on a simulated quality inspection task similar to the SART paradigm (i.e. a vigilance task requiring inhibition of an habitual response) also declines over extended periods. Furthermore, Manly and colleagues [4] reported a time of day effect on overall response inhibition using the SART, such that accurate inhibition of responses on no-go trials was lower during early morning and night as compared to the afternoon and evening times. In contrast, RTs of go trials, assumed to reflect automatic processing, were not modulated by time of day. These studies together reveal that both time on task and time of day produce important effects on executive control during response inhibition tasks. However, to the best of our knowledge, the joint impact of these factors on inhibitory control had not been studied previously.

On the other hand, chronotype refers to individual differences regarding both the preferred time of day to perform activities and sleep timing. Chronotype has a genetic basis [14], it affects the temporal organization of physiological functions and behaviours, and can therefore influence cognitive functioning through the day [9]. There are three main circadian typologies or chronotypes: morning-type (‘larks’), intermediate-type and evening-type (‘owls’) [15]. With regard to evening-type, morning-type people show a 2–4 h advance in circadian phase in variables like subjective alertness, sleep times, core body temperature (CBT) or distal skin temperature (DST) [16]–[20]. Given that DST is closely associated to the CBT rhythm (showing an advanced rhythm phase and inverse temporal curve with maximum values within the sleeping period) [21], DST has been proposed as a reliable circadian index under free-living conditions [22], [23]. Additionally, infraclavicular temperature and the difference between distal and proximal temperatures (distal-proximal gradient, DPG) have been related to the vigilance state. Increments in infraclavicular temperature correlate with slower reaction time in the PVT [24], and DPG increments correlate with short latency of sleep onset [21], [25].

Given the differences in circadian rhythmicity between morning and evening chronotypes, it is natural to expect variations in task performance as a function of both chronotype and time of day. The interaction between chronotype and time of day is referred to as the synchrony effect, and involves better performance for optimal (morning for morning-type and evening for evening-type) as compared to non-optimal times of day [26]–[29]. The synchrony effect has been found in a wide range of executive tasks, for example measuring response inhibition [27], [30], [31], fluid intelligence [32] and set-shifting abilities [33] (for a review see [9]). However, these studies have focused on measures of overall performance (averaged across the whole session), rather than on the evolution of performance across time. Thus, it remained to be tested whether the vigilance decrement during an executive control task is influenced by the synchrony effect.

The aim of the current research was to study the impact of time on task on executive control (vigilance decrement), and to test for the first time whether this decrement changes as a function of time of day and individual differences in chronotype. We used a 20-min long version of the SART task with two response strategy conditions: precision (controlled responding set) vs. speed (automatic responding set). According to research suggesting that controlled but not automatic processes are vulnerable to the synchrony effect [27], [30], we expected the synchrony effect to occur selectively in the precision strategy condition. In contrast, performance in the speed condition inducing an automatic response style should remain relatively stable. Following the cognitive resource theory, we further expected to observe the highest vigilance decrement in the most cognitively demanding condition of the modified SART (i.e., precision strategy), performed at the non-optimal time of day. Finally, we tested whether inhibition performance on SART could be predicted by other measures (RT performance in the PVT and scores in MAAS and ARCES questionnaires [34]) of vigilance.

## Method

### Ethics Statement

Before the experiment all participants signed a consent form approved by the Ethics Committee of the University of Granada. This study was conducted according to the ethical standards of the 1964 Declaration of Helsinki. After the experiment, participants were rewarded with course credits for their collaboration.

### Participants

Forty-four undergraduates from the University of Granada were initially selected to participate according to their score on the reduced scale of Morningness-Eveningness Questionnaire (rMEQ; [35]). A strict selection criterion was used to include in the final sample only participants who confirmed their chronotype after a second administration of the rMEQ at the moment of testing. For this reason, ten undergraduates scoring as intermediate-type (from 12 to 16) in the second administration were not included in the final sample. Scores of selected extremes chronotypes showed a strong consistency between both assessments of circadian typology, *r* = .88, *p<*.001. Data from 5 participants who slept less than 5 hours the night prior to the experiment, 1 participant with extremely low accuracy data due to using a wrong response key during most of the experiment and 1 participant who missed the second experimental session, were excluded from analysis. Finally, the sample was constituted by 27 participants, 13 assigned to morning-type group (mean age: 19 years, range: 18–27, SD: 2.4; 12 females; mean score in the rMEQ: 17.85, range: 17–20, SD: 1.14) and 14 to evening-type group (mean age: 19 years, range: 18–23, SD: 1.4; 13 females; mean score in the rMEQ: 9.64, range: 8–11, SD: 0.84).

### Apparatus and Stimulus

#### Questionnaires

Circadian typology was measured by a validated adaptation of the Morningness-Eveningness Questionnaire [16], standardized to the Spanish population: the reduced scale of Morningness-Eveningness Questionnaire [35]. Scores can range from 4 to 25 in a continuum from low to high morningness. Subjective activation and affect were assessed by a 0–100 visual-analog scale (VAS) developed by Monk [36], where 0 indicated the lowest value (minimum activation/positive mood) and 100 the maximum value for both state indices. The Attentional-Related Cognitive Errors Scale (ARCES; [34]) was used to measure susceptibility to cognitive errors in everyday life arising from lapses of attention. Scores can range between 12 (low predisposition to lapses) and 60 (high predisposition). The Spanish version of the Mindful Attention Awareness Scale (MAAS; [37], see also [38]) was used to assess attentional failures, ranging from very frequent (1) up to occasional attention lapses (6). These two questionnaires correlate with SART performance, respectively with the proportion of accurate inhibitions and RT [34]. Trait impulsivity was measured by the adolescent version of the Barratt Impulsivity Scale, appropriate for undergraduate students (BIS 11-A, [39]) and translated to Spanish [40]. Higher scores on the BIS 11-A mean higher impulsivity. We measured impulsivity as it has been related to eveningness [41].

#### Skin temperature recordings

Body temperature was measured using a temperature sensor (iButton- DS1921H; Maxim, Dallas), which has a temperature range from +15°C to +46°C and 1°C of accuracy with a precision of 0.125°C. Three sensors were respectively placed at the palmar side of the wrist of the non-dominant hand (with a sport band), infraclavicular area on the right chest and external malleolus area of the right foot (with adhesive tape). The sensors were programmed to sample every minute along the experimental session. Note that the total sample and group sizes differed across the recordings of body temperature for technical reasons. Nineteen participants had wrist temperature recordings (9 morning-type, 10 evening-type), 25 participants had foot temperature recordings (11 morning-type and 14 evening-type) and 26 participants had infraclavicular temperature recording (13 morning-type, 13 evening-type).

#### Behavioural Tasks

Experimental tasks were performed on a 15-inch screen PC laptop computer. Programming, administration of tasks and behavioural data collection were controlled by E-prime software [42].

The *Psychomotor Vigilance Task* (PVT) is a 10-minute simple reaction time task that provides a measure of the overall level of participant’s vigilance [43]. In the current version, a red circumference was presented in every trial at the centre of the screen (9.5 degrees of visual angle at a viewing distance of 60 cm) over a black background. After a random interval, ranging from 2 to 10 seconds, the black circle started to fill up in red and participants had to press as quickly as possible the space bar on the keyboard with the index finger of their dominant hand. After the participant’s response, feedback about the RT in that trial was displayed in the screen for a second. Otherwise, a feedback message was provided on missed or anticipated responses. Then, the next trial began. The task lasted 10 minutes, which on average led to 88 trials.

The *Sustained Attention to Response Task* (SART), as in the original go no-go task developed by Robertson and cols. [12], requires participants to respond as quickly as possible to single digits randomly ranging between 1 and 9, unless the digit 3 was presented, to which they had to inhibit response (no-go trial). Stimuli appeared in white colour over a black background at the centre of the computer screen in one of five possible font sizes (48, 72, 94, 100 and 120 point, Times New Roman) that changed randomly on every trial (from 1.15° to 2.77°). A blank screen was presented for 50 ms followed by a digit that remained on the screen until the participant’s response. If no response was made within 1200 ms, the next trial began. Each experimental block was composed of 200 go trials (5 font sizes × 8 digits × 5 trials) and 40 no-go trials (5 font sizes × 1 digit × 8 trials), leading to a no-go proportion of 0.17.

We used a modified version of the SART, in which the main difference concerned the manipulation of the participant’s style of responding (“strategy”), by providing two different instructions across blocks of trials (see Figure 1). In the accuracy strategy condition, participants were instructed to prioritize accurate over fast performance, hence assuring correct response inhibition in no-go trials. In the speed strategy condition participants were instructed to prioritize fast over accurate performance, hence assuring fast responses to go trials. In order to make sure that each strategy condition was followed, the digit colour changed to provide feedback on line when the criteria for speed and accuracy were not met.

These criteria were established on the basis of the results observed in a previous pilot experiment. In the pilot experiment, participants were assigned to precision or speed strategy groups following a median split procedure based on their average accuracy in no-go trials (M = .71). The analyses showed a significant interaction between time of day, chronotype and strategy, *F* (1, 32) = 4.77, *p = *.03, only in the precision strategy (n = 18; mean RT: 406 ms, SD: 9.9; mean accuracy: 80%, SD: 0.02) but not in the speed strategy group (n = 18; mean RT: 364 ms, SD: 9.9; mean accuracy: 56%, SD: 0.02; *F <*1). These findings suggest that the precision strategy group followed a controlled task set to avoid errors while the speed strategy group applied a more automatic response style.

Therefore, in the accuracy strategy digits were presented in red when the average correct response rate in no-go trials was below 0.71. In that case, participants were instructed to take more time to respond more carefully. In the speed strategy, digits appeared in yellow when both the average RT was above 440 ms and accuracy rate in no-go trials was not below 0.45. That is, participants had to increase response speed when the digit turned yellow. Therefore, digits presented in white indicated adequate performance according to the strategy condition of the current block. In addition, the participants were informed about mean RT and accuracy at the end of each block, during the allowed rest. The task was composed of one practice block and 8 experimental blocks. There were 4 blocks for each strategy condition, and they were presented in alternating runs starting with the precision strategy condition. Variations in performance across these four blocks served to study the vigilance decrement.

### Procedure

Participants completed the rMEQ and the BIS 11-A before the laboratory sessions. Next, they carried out a 1-hour laboratory session twice, at 08∶00 h and 20∶30 h, under dim light conditions (<8 lux). The two sessions were separated by one week, in which participants were instructed to follow their habitual schedule. Therefore, in the present study we used a time of day protocol, whereby morning- and evening-type participants were tested at two different times of day (optimal vs. non-optimal). This paradigm is sensitive to fluctuations in high-order cognitive processes [9], [15] and allows testing under ecological, everyday-living conditions [44]. The order of sessions was counterbalanced across participants within each experimental group (7 out of 13 participants of morning-type group and 7 out of 14 participants of evening-type group completed the first session in the morning). When the participant arrived at the laboratory, temperature sensors were placed at three different locations of the body. Then, participants completed the ARCES and the MAAS questionnaires (in the first and second session respectively), and reported about sleep duration, psychiatric and sleep disorders, consumption of stimulants, subjective activation and affect. Afterwards, the PVT was administered to obtain an objective index of vigilance that is sensitive to the synchrony effect. Finally, participants completed the main task, the SART, for 20 minutes approximately.

### Design and Statistical Analysis

The rMEQ scores and chronological age were analyzed by one-way analysis of variance (ANOVA) to test for possible differences between morning-type and evening-type chronotype groups. Skin temperature data from each sensor location were analyzed by averaging values within the first 10 minutes (i.e., during the PVT). The distal (wrist) to proximal (infraclavicular) temperature gradient (DPG) was also computed for every participant at each time of day condition.

Body temperature, sleep duration, sleep onset and offset times and time awake before testing, subjective affect, and RT and accuracy in the behavioural tasks, were submitted to separate ANOVAs. We used a mixed-design ANOVA of 2 (Chronotype: Morning-type, Evening-type)×2 (Time of Day: Morning, Evening), with chronotype as a between participants factor and time of day manipulated within participant. The PVT analysis excluded the first five trials, which were considered as practice, and trials with RTs below 100 ms and longer than 1000 ms (0.09% rejected).

Similarly, the RT analysis of SART excluded trials with RT below 100 or above 1000 ms (0.007% excluded), practice trials (i.e., trials from the practice block and the first five trials of every experimental block, which were considered as warm-up trials) and incorrect trials (i.e., responses in the no-go condition). The accuracy analysis of SART computed the proportion of correct responses in the no-go condition (i.e. responses correctly inhibited). The SART analysis further included Strategy (Accuracy, Speed) and Block (from 1 to 4) as within participants’ factors. Mauchley’s test showed no violation of sphericity for the main effect of block and interactions with the block factor (all *ps >*0.40). To study the role of different response strategies, strategy was manipulated within-participants, so that different blocks emphasized accurate response-inhibition or speeded response style. Moreover, the vigilance decrement in performance was analysed by including block as within-participants factor. When the effect of block was significant, polynomial trend analyses (linear, quadratic and cubic trends) were performed to characterize how executive control evolved along time on task. Furthermore, simple linear correlations were calculated between self-report questionnaires (ARCES and MAAS), performance on the PVT and inhibitory performance on the precision strategy condition for each participant at both morning and evening sessions.

## Results

### Demographic Data

The analysis conducted on the rMEQ scores confirmed significant differences between our chronotype groups, *F* (1, 25) = 455.31, *p<*.01, ŋp^2^ = 0.95, with higher morningness scores in the morning- vs. evening-type group (see table 1). In contrast, age did not differ between groups, *F <*1. Sleep duration (the night before experiment) was longer in the evening (M: 7.6 hours, SD: 0.28) than in the morning session (M: 6.1 hours, SD: 0.14), *F* (1, 25) = 29.53, *p<*.01, ŋp^2^ = 0.54. Importantly, however, no differences were observed between morning-type and evening-type groups in sleep duration, *F <*1, and the interaction between time of day and chronotype was not significant either, *F <*1, therefore confirming similar sleep duration between groups. In contrast, the analysis of waking duration prior to sessions showed a marginally significant interaction between testing time and chronotype, *F* (1, 25) = 4.16, *p = *.07, ŋp^2^ = 0.12. In particular, chronotype groups differed in time awake before the evening session, *F* (1, 25) = 4.16, *p = *.05, with more awake hours in morning-type (M = 11.88, SD = 0.41) relative to evening-type group (M = 10.71, SD = 0.40), but no differences were found for the morning session (*F <*1). The sleep onset time analysis only showed a main effect of time of day, *F* (1, 23) = 11.55, *p<*.01, ŋp^2^ = 0.33, with a latest onset in the evening (M = 1∶36, SD: 0.30) than in the morning session (M = 00∶22, SD = 1.53). Similarly, sleep offset time showed a significant time of day effect, *F* (1, 23) = 40.70, *p<*.01, ŋp^2^ = 0.64, with earlier waking up time in the morning (M = 6∶38, SD = 0.11) than in the evening session (M = 9∶02, SD = 0.27). No significant interactions or chronotype effects were found for both sleep onset and offset analysis (all *ps >*0.09).

**Table 1:** Mean and standard deviation (between brackets) for demographic data according to chronotype group.

Group characteristics	Chronotype Groups		p values
	Morning-type	Evening-type	
Sample size	13	14	
rMEQ	17.85 (0.28)	9.64 (0.27)	0.01
Age	19.23 (0.55)	18.71 (0.53)	0.5
Sleep duration before morning session (in hours)	6.21 (0.21)	5.96 (0.20)	0.4
Sleep duration before evening session (in hours)	7.54 (0.41)	7.68 (0.39)	0.8
Hours awake in morning session	1.41 (0.16)	1.43 (0.16)	0.9
Hours awake in evening session	11.88 (0.41)	10.71 (0.40)	0.05
Sleep onset before morning session (hh:mm)	00∶25 (0.27)	00∶22 (0.23)	0.9
Sleep onset before evening session (hh:mm)	1∶05 (0.45)	2∶08 (0.46)	0.13
Sleep offset before morning session (hh:mm)	6∶36 (0.19)	6∶43 (0.19)	0.7
Sleep offset before evening session (hh:mm)	8∶37 (1.22)	9∶30 (0.42)	0.16
Smokers	1	2	
Consumption of coffee/tea	3	3	
ARCES	29.18 (2.13)	34.14 (1.89)	0.1
MAAS	4 (0.21)	4 (0.21)	0.9

Furthermore, 2 out of 13 morning-type and 3 out of 14 evening-type participants reported caffeine consumption at least 5 hours before their non-optimal testing time. One morning-type and 2 evening-type participants were smokers.

Analysis of scores in the BIS-11A (n = 19; 8 M-type, 4 of them completed the first session in the morning; 11 evening-type, 5 evening-type assigned to the morning in the first session) revealed higher trait impulsivity in evening-type as compared to the morning-type group, *F* (1, 17) = 6.50, *p = .*02, ŋp^2^ = 0.28 (Table 1). Thus, the BIS-11A score was later used as a covariate to control for group differences in trait-impulsivity. Analyses on ARCES (n = 25) and MAAS (n = 27) showed no effect of group (all *ps >*.10).

### Skin Temperature Recordings

The infraclavicular temperature analysis showed a significant interaction between chronotype and time of day, *F* (1, 24) = 4.96, *p = *.03, ŋp^2^ = 0.17. Planned comparisons revealed that the evening-type group had higher temperature values in the evening (M: 33.81°C, SD: 0.31°C) than in the morning (M: 32.69°C, SD: 0.31°C) session, *F* (1, 24) = 8.89, *p<.*01, while the morning-type group showed no differences (*F<*1). Wrist temperature only showed a main effect of time of day, *F* (1, 17) = 4.36, *p = *.05, ŋp^2^ = 0. 20, with higher temperature in the evening (M: 33.23°C, SD: 0.23°C) than in the morning (M: 32.68°C, SD: 0.35°C) session. No significant main effects or interactions were observed in the analysis of right foot temperature (all *ps >*.18).

The DPG analysis showed a significant interaction between time of day and chronotype, *F* (1, 16) = 4.88, *p* = .04, indicating a synchrony effect. Morning chronotypes showed the most negative DPG value in the morning (M: −1.47, SD: 0.9) although the difference was not significant with respect to the other conditions (all *ps >*.10).

### Subjective Activation and Mood States

The subjective activation analysis showed a significant interaction between chronotype and time of day, *F* (1, 25) = 7.20, *p = *.01, ŋp^2^ = 0.22. Planned comparisons showed that evening-type participants reported higher activation in the evening (M: 49.28, SD: 4.89) compared to the morning session (M: 35.21, SD: 5.66), *F* (1, 25) = 5.67, *p = *.02, while the time of day effect followed an opposite trend for the morning-type group, although it did not approach significance (M: 56.56, SD: 5.87 vs. M: 47.77, SD: 5.07 for morning vs. evening sessions, respectively; *F* (1, 25) = 2.05, *p = *.16). In addition, significant differences in self-reported activation between chronotypes were found for the morning session, *F* (1, 25) = 6.85, *p = *.01, but not for the evening session (*F <*1).

Regarding subjective affect, both chronotypes reported more positive affect at their optimal time of day (chronotype×time of day: *F* (1, 25) = 4.36, *p = *.05, ŋp^2^ = 0.15). In particular, the evening-type group showed less positive affect in the morning (M: 66.70, SD: 4.99) than in the evening session (M: 74.71, SD: 4.45), *F* (1, 25) = 5.24, *p = *.03, but the morning-type group did not show significant differences between both testing times (*F <*1; M: 73.52, SD: 5.18 vs. M: 71.00, SD: 4.62 for morning vs. evening sessions, respectively).

### Psychomotor Vigilance Task (PVT)

**Figure 2:**
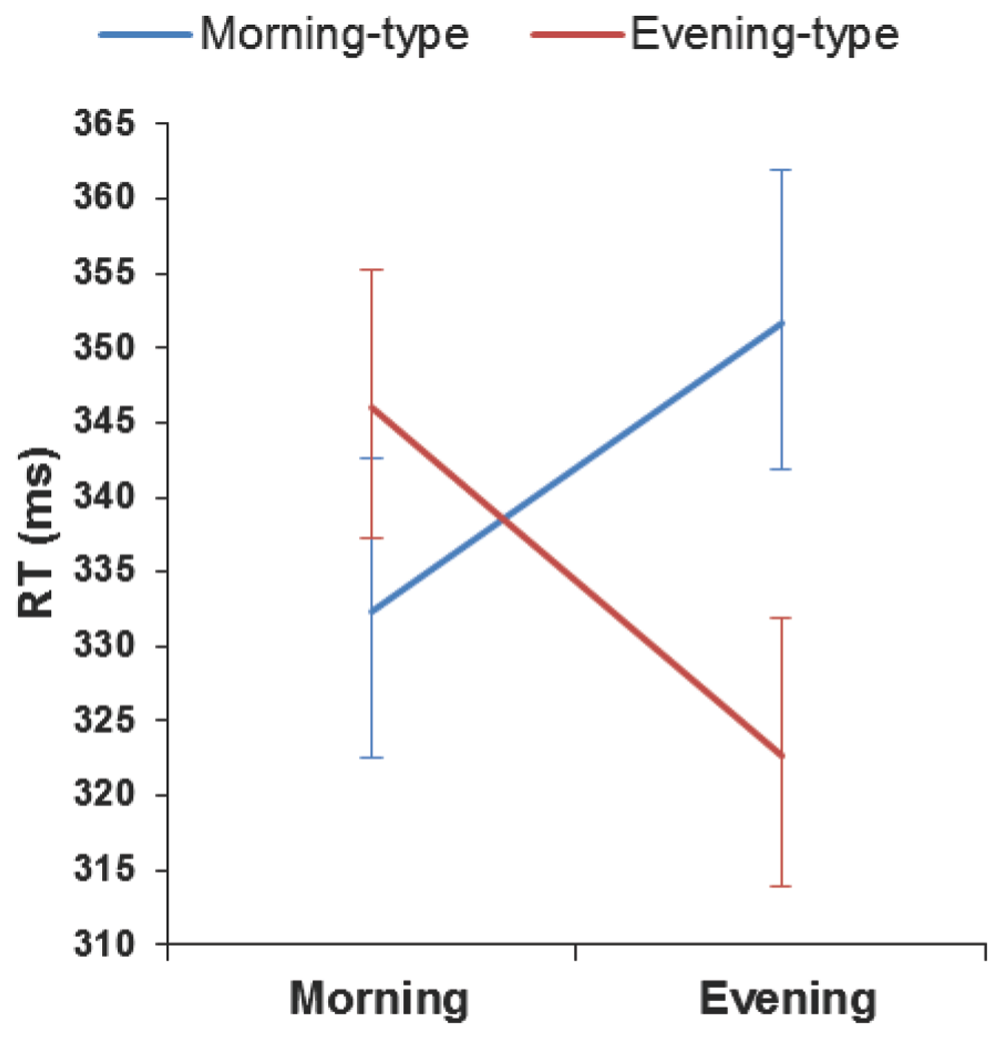
Mean reaction times on the PVT for both chronotypes depending on time of day. Each chronotype responded fastest at their optimal time of day and slowest at their non-optimal testing time. Vertical bars denote +/− standard error of the mean.

As Figure 2 shows, the RT analysis showed a clear synchrony effect (chronotype×time of day: *F* (1, 25) = 11.71, *p<*.01, ŋp^2^ = 0.32). In the morning-type group, RTs were faster in morning vs. evening sessions, *F* (1, 25) = 4.63, *p = *.04. In contrast, the evening-type group showed slower RTs in the morning vs. evening session, *F* (1, 25) = 7.29, *p = *.01. Moreover, in the evening session, RTs were faster for evening-type than for morning-type groups, *F* (1, 25) = 5.17, *p = *.03. However, no difference was observed in the morning session, *F <*1.

### Sustained Attention to Response Task (SART)

#### Reaction Times

The ANOVA on the mean RT showed a significant effect of Strategy, *F* (1, 25) = 84.33, *p<*.01, ŋp^2^ = 0.77, with slower RT in the precision condition (M: 385 ms, SD: 6.86 ms) than in the speed condition (M: 336 ms, SD: 5.56). The remaining main effects and interactions did not reach statistical significance (all *ps >*.12).

#### Accuracy

The main effect of strategy was significant, *F* (1, 25) = 92.50, *p<*.01, ŋp^2^ = 0.78, with higher accuracy in the precision strategy (76%) than in the speed strategy condition (57%). The main effect of block, *F* (3, 75) = 15.04, *p<*.01, ŋp^2^ = 0.37, revealed impaired response inhibition with increasing time on task. Further analyses replicated the typical vigilance decrement, which followed a linear trend, *F* (1, 25) = 34.71, *p<*.01 (the quadratic trend was not significant, *p>*.2). Most relevant for the current research, the ANOVA showed a significant interaction between chronotype, time of day and block, *F* (3, 75) = 2.94, *p = .*038, ŋp^2^ = 0.10. This interaction was better qualified by considering the strategy factor, as suggested by a marginally significant interaction between strategy, chronotype, time of day and block, *F* (3,75) = 2.60, *p = .*058, ŋp^2^ = 0.09. Differences between both strategy conditions were tested by hypothesis-driven planned comparisons [45]. As we predicted, the interaction between chronotype, time of day and block (linear trend) was significant only for precision strategy, *F* (1, 25) = 5.18, *p = *.03, but not for speed strategy, *F <*1.

**Figure 3:**
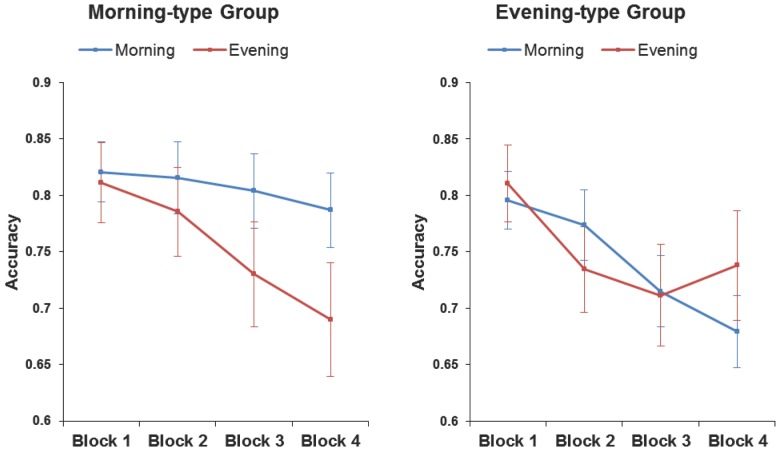
Accurate responses as a function of chronotype, testing time and block for the precision strategy condition. Each chronotype showed marked performance decrements at the non-optimal testing time. Vertical bars denote +/− standard error of the mean.

Further analyses of the precision strategy condition revealed that the linear decrement in vigilance was only significant when the groups performed the task at their non-optimal time of day according to chronotype (see Figure 3). That is, in the morning-type group, there was an interaction between time of day and block (linear trend contrast), *F* (1, 25) = 4.20, *p = *.05, such that correct response inhibition linearly declined with time on task in the evening session, *F* (1, 25) = 11.09, *p<*.01, but not in the morning session (*p>*.24). In the evening-type group, although the interaction between time of day and the linear trend of block was not significant, *F* (1, 25) = 1.33, *p = *.25, further analyses clearly showed a linear decrement on accuracy in the morning session, *F* (1, 25) = 19.90, *p<*.01, while the block effect in the evening session was better characterized by a quadratic trend, *F* (1, 25) = 4.86, *p = *.04, where accuracy decreased until the third block and then remained stable), rather than a linear trend, *F* (1, 25) = 3.94, *p = *.058.

#### Analysis of covariance in accuracy

Since evening-type showed significantly higher impulsivity than morning-type group (see Demographic Data results), we used BIS-11A scores as a covariate to control for a possible confound of trait-impulsivity in our main manipulation. This covariance analysis confirmed that the interaction between strategy, chronotype, time of day and block remained significant, *F* (3, 48) = 2.88, *p = .*04, after controlling for trait-impulsivity, which suggests that our main effects can be attributed to the chronotype manipulation rather than just by differences in impulsivity.

### Correlation Analyses

**Figure 4:**
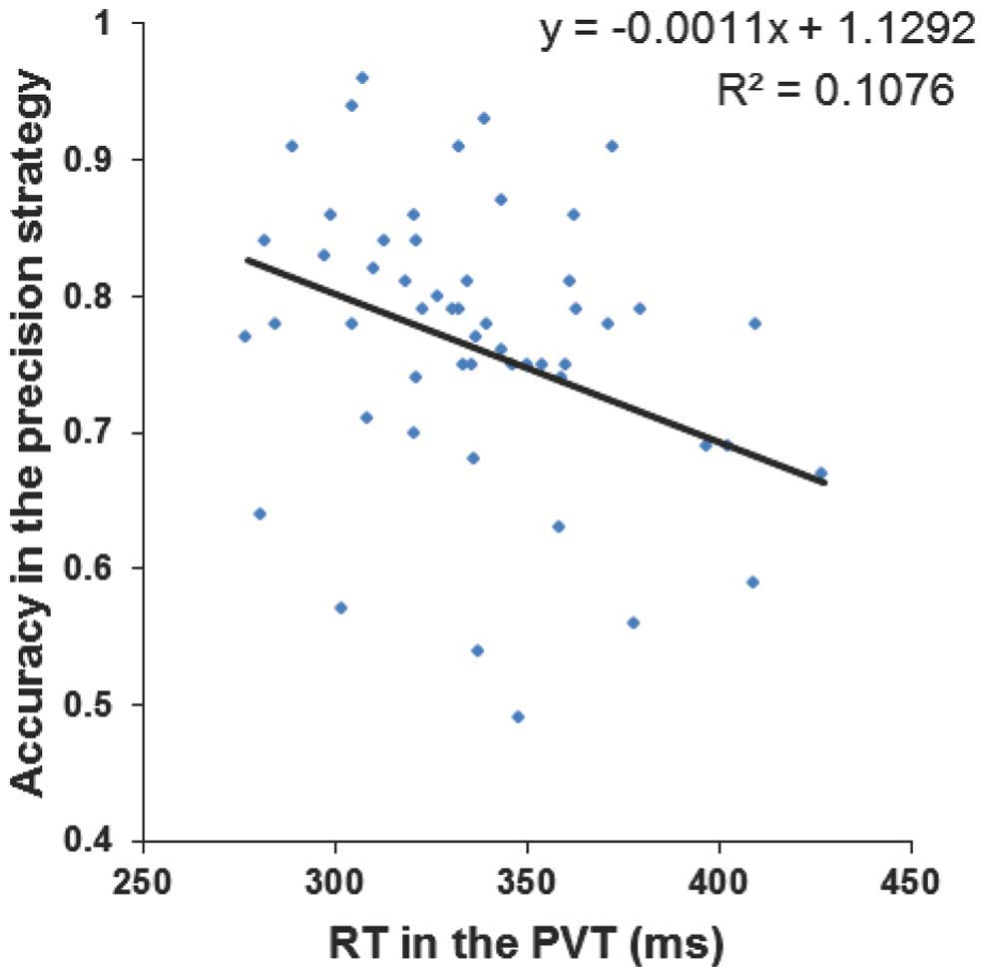
Correlation between RT in the PVT and accuracy on SART for the precision strategy condition.

Analysis of simple linear correlations between scores of the MAAS and ARCES scales, RT performance in the PVT and accuracy performance on SART for precision strategy were conducted (the BIS score was not included due to missing data for 8 participants). The main finding was that inhibitory performance on SART was correlated with performance on the PVT (*r = *-.33, *p = *.01). Thus, optimal vigilance states were associated with more successful response inhibition (see Figure 4). Accuracy in the SART positively correlated with the MAAS scale (*r = *.47, *p<*.01), such that participants reporting more infrequent attentional lapses showed better response inhibition. The ARCES scores did not correlate with performance on SART (all *ps >*.09).

## General Discussion

The main aim of the present study was to investigate the impact of both time of day and circadian typology (i.e., the synchrony effect) on the vigilance decrement in inhibitory control during a modified version of the SART [12]. Participants completed the task at morning and evening sessions in a counterbalanced order within morning-type and evening-type groups. According to previous research [4], [27], we predicted that controlled processing would be more vulnerable than automatic processing to the influences of time of day and chronotype. The synchrony effect would therefore be most evident in the precision strategy condition demanding a controlled task set. Since the ability to maintain successful response inhibition over time also demands resources of cognitive control [46], we expected to find the largest vigilance decrement at the non-optimal time of day for each chronotype.

Two findings supported our main prediction. First, the synchrony effect was selectively found in no-go accuracy performance (assumed to index executive control) rather than on go RTs, indexing more automatic processing [4], [12]. Second, the synchrony effect was present in the precision strategy condition, which demanded a controlled response set, but it was absent in the speed strategy condition, which induced an automatic response set. In the precision strategy condition, inhibitory control of the morning-type group decreased gradually over time on task only in the evening, but not in the morning. In contrast, evening-type participants showed a more marked performance decrement in the morning than in the evening session, in which accuracy decreased but remained stable over the final testing period.

Further analyses of the synchrony effect in our objective and subjective measures revealed that response inhibition was most effective when participants performed the SART in their optimal vigilance state. Optimal vigilance was specifically indexed by faster RTs in the PVT [3], higher levels of subjective activation [36], and lower DPG [25] and infraclavicular temperature [24] when the session matched the optimal testing time of each chronotype.

The link between vigilance state and executive control was further supported by a significant negative correlation between RT performance in the PVT and accuracy in the SART, whereby the optimal vigilance state as indexed by fast RTs was related with enhanced inhibitory control. Therefore the PVT could be a useful tool to predict performance on tasks demanding executive control. Furthermore, high MAAS scores (i.e. subjective experience of fewer lapses of attention; [38]) were also found to predict inhibitory performance, which to our knowledge had not been reported previously [34].

These findings therefore reveal a close interplay between the ability to remain vigilant and the executive control process involved in response inhibition [47]. According to conceptions of vigilance as an active, resource-demanding process [2], [46], [48], [49] we conclude that performing at non-optimal times of day jeopardizes the engagement of necessary resources to maintain appropriate inhibitory control throughout the task. In contrast, testing at the preferred time of day may mitigate the decline in performance across time on task. Neuropsychological and imaging studies have related SART performance to a right lateralized brain network involved in vigilance and executive control, with emphasis on the right prefrontal cortex [47], [50], [51]. A recent fMRI study additionally showed a synchrony effect on anterior brain areas involved in executive control as demanded by a Stroop interference task [52]. Our findings and the above studies hence support the notion that vigilance plays a central role in executive functioning related to inhibitory control.

The current research further highlights the role of individual differences in chronotype regarding the ability to maintain executive control under free-living conditions. Thus, detailed analyses of the synchrony effect in our main measures revealed that time of day effects were less evident in morning-type than evening-type participants. In fact, only the morning-type group was able to prevent completely the vigilance decrement at the optimal time of day. This result is consistent with our previous study on simulated driving, reporting that only the morning-type group showed stable performance over time on task, which was not affected by time of day [53]. Some authors have interpreted this result in terms of personality factors [54], associating evening-type participants to low conscientiousness, high impulsivity, higher sensation seeking and reduced vigilance (reviews by [55], [56]). Indeed, differences in impulsivity might have mediated our effects of chronotype [41]. However, the analysis including trait impulsivity as covariate suggested that our findings cannot be explained just in terms of impulsivity, but they can be attributed to individual differences in chronotype.

An alternative explanation considers the difference in sleep homeostatic dynamics between chronotypes. Research relating eveningness with irregular sleep/wake habits and increased need for sleep than morningness, which shows higher sleep efficiency [57], [58], can help to explain the robustness of behaviour in morning chronotypes. Although our participants did not report to have sleep problems, further information about sleep quality (e.g., Pittsburgh Sleep Quality Index – PSQI) in our groups should have been recorded to test this hypothesis.

Likewise, the current research presents other limitations that should be addressed in future research. First, this study was designed from a time of day rather than a circadian physiology perspective. Recording of sleep/wake habits by sleep diary and circadian markers (e.g., actimetry) during the week before the experiment would be necessary to warrant interpretation of our time of day effects in terms of pure differences in circadian phase. Although body temperature recorded at the beginning of each session showed a synchrony effect (suggesting that chronotypes were at different circadian phases in the two sessions), we acknowledge that only two time points cannot provide clear information on whether circadian rhythms of our sample were really entrained to our specific testing times.

Moreover, our study focused on cognitive testing under free-living rather than strict laboratory conditions, which involved no restriction of stimulants or careful control of other masking influences such as feeding schedule. In fact, participants followed their natural habits during the course of the study. Since the proportions of smokers and coffee drinkers in our sample were relatively small and matched for morning-type and evening-type groups, it is likely that any masking influence of these factors on performance were balanced and did not preclude the current finding of clear interactions between time of day and chronotype.

In future studies it will also be interesting to adapt testing times to individual sleep/awake patterns [59]. In the current study, several participants in both groups slept about six hours, which could be considered as sleep restriction, and evening-type chronotypes were tested earlier than their usual preferences in the morning session. However, both extreme chronotypes recruited in the study reported similar sleep duration, and similar sleep timing before the morning session, so that chronotype effects on the performance during the morning session cannot be just due to restricted sleep duration selective of the evening-type group. As already mentioned, nevertheless, we cannot rule out explanations based on greater sleep need and debt or increased dissipation time of sleep inertia during working days reported in evening-type people [58], [60].

Addressing the abovementioned limitations will surely improve our current understanding of the neurophysiological mechanisms underlying our main behavioural result. Our findings observed under free-living conditions are consistent with previous research using more controlled protocols (constant routine or forced-desynchrony) and reporting circadian rhythmicity of executive functions [5], [6] (but see [61]). However, the previous research did not address the vigilance decrement in cognitive performance.

Research on decrements in vigilance during everyday tasks demanding executive control is crucial for ergonomics, since it can lead to serious consequences for safety in transport and work. The current study provides, for the first time, empirical evidence indicating that the vigilance decrement of executive functioning depends on the interaction between circadian typology and time of day factors. The amount of necessary cognitive resources to maintain adequate executive function over time on task could be regulated by a complex interplay between circadian and homeostatic influences underlying time of day and chronotype effects. The current study provides implications concerning the importance of considering chronotype and time of day when scheduling tasks demanding sustained executive control, not only in clinical and research contexts of cognitive testing, but also in everyday and work-related situations where optimal cognitive functioning can be critical.
